# Editorial: Supporting families in the first 1,000 days of life – a balancing act

**DOI:** 10.1111/jcpp.70041

**Published:** 2025-09-08

**Authors:** Maartje P.C.M. Luijk, Tessa J. Roseboom

**Affiliations:** ^1^ Erasmus School of Social and Behavioural Sciences Erasmus University Rotterdam Rotterdam The Netherlands; ^2^ Department of Epidemiology and Data Science Amsterdam University Medical Center Amsterdam The Netherlands

**Keywords:** Early‐life experiences, parenting, infancy, social policy, child development

## Abstract

From the moment of fertilization, human development takes a phenomenal pace. In no other period of life are more biological and developmental milestones met than in the first 1,000 days after conception. All organs and systems are formed, and children start to discover the world, learn whom to trust and where to find comfort in times of distress. Evidence from biological, psychological, social and economic research shows that the environment in the first 1,000 days significantly impacts a person's ability to reach their full potential. Children who grow up in unpredictable and unsafe environments often struggle with the consequences for the rest of their lives. Investments in this critical period of human development have proven to be the most cost‐effective way to improve lifelong health and well‐being. Therefore, this period has gained interest both in political debate and society at large. In this contribution, we demonstrate that while the focus on the first 1,000 days is scientifically sound and historically grounded, it is time to reflect on its societal impact. We focus on its unintended negative consequences for parental well‐being. Evidence for the importance of the first 1,000 days should drive collective action rather than reinforce individual blame. Parenting is not just a private matter; governments have a duty to provide parents with the resources to give their children the best start in life.

## Why early‐life experiences have lasting societal effects

The importance of the early environment for human health and well‐being has been demonstrated by a wealth of scientific studies (such as reviewed in Gluckman, Hanson, Cooper, & Thornburg, 2008). Studies of humans exposed to the 1944–45 Dutch famine in utero show lasting effects of war‐related stress and acute undernutrition on physical and mental health (Bleker, de Rooij, Painter, Ravelli, & Roseboom, 2021). These findings support the Barker hypothesis (Barker & Osmond, 1986), which links inadequate prenatal and early postnatal nutrition to increased risk of chronic diseases in adulthood. Moreover, animal experiments confirmed causality and revealed the epigenetic basis of early‐life exposures causing lifelong health vulnerabilities, although translating findings from animal models to humans requires caution. Experiments in rats demonstrated that the behaviour of mother rats towards their pups affected the offspring's brain physiology and sensitivity to stress through epigenetic mechanisms (Fish et al., 2004) and these effects transferred to the next generation. Studies on the impact of growing up in orphanages, that is, environments characterized by deprivation, show that institutionalized children exhibit delays in all developmental areas (e.g. Smyke et al., 2007). Despite the large body of evidence from human and animal studies on the impact of early‐life stressors on offspring development, the long‐term effects vary considerably and may be modest at the individual level (Dunn et al., 2018).

Yet, the societal relevance of the impact of adverse exposures in early life was revealed by one of the world's longest‐running multidisciplinary human development research projects, the Dunedin study (Richmond‐Rakerd et al., 2020). Analyses in New Zealand, and replicated in Denmark, showed that the majority of societal costs across multiple domains (health, crime, unemployment and insurance claims) were incurred by individuals who, as children, had experienced early‐life stressors, including growing up in poverty, in unsafe homes, or with a parent with severe mental health problems. Their early environments were marked by scarcity, violence and unpredictability, which hindered psychological, physical and brain development. As adults, they were at higher risk for learning and behavioural difficulties; mental health problems were more prevalent, and they were less able to participate in the labour market. The results of longitudinal studies, animal studies and natural experiments in various settings across the globe show that the early environment in which children develop affects current‐day societal problems.

## Investing in families: The role of governments in early support

Nobel Laureate Heckman et al. (Heckman, 2006) have shown that every dollar invested in early development, for example, through healthy food and cognitive stimulation, yields a multiple dollar profit. Investments in early life lead to better academic performance, greater labour market participation, less dependency on social benefits, and fewer costs for society. Other economic evaluations show that investments in longer and more flexible parental leave schemes translate into higher incomes for the next generation. Although early investment is widely supported, a more nuanced perspective cautions against oversimplifying its implications, recognizing that developmental plasticity continues throughout childhood and adolescence. Moreover, the effects of early interventions may fade out, and support remains valuable across later stages of development. Together, these findings suggest that investing early is a promising strategy, and governments around the globe are increasingly recognizing the value of this foundational period.

Three overarching societal trends may contribute to the complexities of modern parenting: the erosion of traditional support networks, the pervasive influence of perfectionism and the ambiguity surrounding typical child development.

Next to the biological, psychological and social scientific evidence and the economic rationale, there is a third compelling argument to invest in the first 1,000 days of life, the justice argument based on the Convention on the Rights of the Child. The Convention explicitly states that governments should support parents in their child‐rearing responsibilities: ‘Parents or, as the case may be, legal guardians, have the primary responsibility for the upbringing and development of the child. The best interests of the child will be their basic concern. (…) States Parties shall render appropriate assistance to parents (…) in the performance of their child‐rearing responsibilities and shall ensure the development of institutions, facilities and services for the care of children’. (United Nations General Assembly [UNGA], 1989, art. 18). In other words, governments have an important responsibility in supporting parents to raise their children. This support can take many forms, such as policies on parental leave, high‐quality child care or accessible preventive health care.

Crucially, the Convention underscores that governments must support parents in their child‐rearing responsibilities. This means that parenting is not solely a private matter, but one in which governments have a clear duty to provide appropriate support structures, ensuring that parents have access to the resources they need to give their children the best possible start in life.

## Policies, *not* individual choices, shape long‐term health outcomes

Although the evidence for the importance of the first 1,000 days is strong, and initiatives aimed at improving early childhood development are well‐intended, it is not easy to strike the right balance in messages in policy and practice. Too easily, the evidence from scientific studies is described in a deterministic way, reducing complex developmental processes to simple cause‐and‐effect explanations. This frequently leads to an implicit – or even explicit – blaming of parents. Such messages ignore the multifactorial nature of early‐life vulnerabilities, the cumulative effects of broader environmental factors, and society's responsibility for the well‐being of families.

A striking example is a recent study on the long‐term health effects of government‐imposed sugar rationing during the first 1,000 days of life (Gracner, Boone, & Gertler, 2024). As part of broader wartime and post‐war food policies, governments controlled sugar distribution, limiting access for certain populations. By examining historical data, the study assessed how these state‐enforced restrictions during the first 1,000 days influenced metabolic and cardiovascular health in adulthood. The study gained widespread media attention, yet most news items misleadingly suggested that women who consume too much sugar during pregnancy or infancy would harm their child's health. In reality, the research examined government‐imposed sugar rationing, highlighting how state policies, *not* individual choices, shaped long‐term health outcomes. The message should have been about the influence of governments on public health rather than placing responsibility on individual mothers. Instead of fostering a collective understanding of societal influences on child development, the dominant narrative shifted responsibility onto individual parents, reinforcing a cycle of individual blame rather than addressing systemic factors.

## The pressure to ‘get it right’

Influenced by science and policy, the current narrative on the importance of the first 1,000 days can unintentionally heighten pressure on parents. Parents are very much aware of the importance of their influence on their child's development, and campaigns that highlight the importance of a good start may convey a message to parents that they need to work harder, do better, and that any ‘mistake’ will have lifelong consequences. This creates an unwanted burden with potentially counterproductive effects.

A recent report by the Office of the Surgeon General (2024), highlights the alarming rise in stress and declining mental health among caregivers. Half of all parent's experience intense exhaustion, and 20% of all parents show symptoms of parental burnout. Parental burnout is a condition of overwhelming exhaustion related to parenting, loss of pleasure in the parenting role and emotional distancing from the children and now affects 2%–9% of parents in individualistic cultures, such as the Netherlands and the United States (Roskam et al., 2021). Because parents cannot resign from their role as a parent, parental burnout is more damaging than job burnout and leads to pronounced societal costs and personal damage, including risk for depression, suicidality, substance use and child maltreatment. Three overarching societal trends may contribute to the complexities of modern parenting: the erosion of traditional support networks, the pervasive influence of perfectionism and the ambiguity surrounding typical child development. Parenting, once a community effort, has become an individual responsibility, and many parents lack community support. The prevailing ethos of self‐reliance discourages parents to reach out to friends, family or community members for help. In the Netherlands, the majority of parents feels ashamed to ask for help, and a global study showed that parental burnout is most prevalent in Western countries characterized by high individualism (Roskam et al., 2021). Second, societal pressures emphasizing perfectionism in parenting exacerbates parental stress. Intensive parenting, that is devoting much time and energy to the child, has become mainstream. The pressure to ‘get it right’ places a large burden on parents, it undermines parental confidence and contributes to feelings of inadequacy. And third, despite access to abundant information on child development, parents often feel uncertain about what is ‘normal’. The myth of normality, that is idealizing averages while ignoring natural variation, fuels uncertainty. Common child behaviours like crying, are often labelled as problematic, and reason to seek professional help. This strains youth care services, shifting limited resources to preventable cases and reducing support for families with more severe problems. This increases both individual and societal costs.

## The history of ‘getting it right’: How pressure on parents developed over time

To understand how attention for the importance of early child well‐being developed, we have to look back in time. At the end of 19th century, infant and child mortality declined rapidly due to enhancements in public health, medical knowledge and hygiene. This progress fuelled hopes for similar improvements for mental health, inspiring the Mental Hygiene Movement, which spread to Europe in the 1920s (Van der Horst, Van Rosmalen, & Van der Veer, 2024). The movement emphasized early prevention, while psychoanalysis, emerging around the same time, attributed adult mental problems to childhood experiences. This placed the responsibility of later well‐being on the shoulders of parents. Together with Bowlby's work on mother–child separation and the development of attachment theory, these ideas reinforced a strong belief in the formative power of early upbringing. Since mothers were seen as primary caregivers, they were often held responsible for how their children would turn out later in life and blamed for any negative outcomes (Van der Horst et al., 2024). These ideas were not limited to child psychiatry and psychology; they also impacted public policy and child‐rearing practices.

The emphasis on individual parental responsibility for child well‐being persisted, and another example is found in the 1990s. Brain research showed the potential of enhancement of brain capacity in children. This idea gained a prominent place in child advice literature and discourse and had major influence on parents. It added to the idea that child development can be ‘optimized’ if parents work hard enough, using stimulating toys, monitoring apps and training by experts. It also added to a ‘warning’ perspective; the idea that developmental problems could be prevented or solved by the way parents nurtured the developing brains of their infants (Macvarish, 2023). The optimizing and warning perspectives underline the pressure on individual parenting choices and parents' responsibility for well‐being. Over time, the belief in the formative power of early childhood, reinforced by scientific discoveries, has contributed to a growing emphasis on parental responsibility for optimal child development. The dual message of opportunity and risk has intensified pressure on parents, framing child‐rearing not only as a personal responsibility but also as a high‐stakes endeavour where every decision feels crucial.

## Changing the narrative: Supporting families by strengthening societal systems

There is a long history of society blaming parents, and specifically mothers, for the ill health of their children. Unfortunately enough, scientific findings have reinforced perspectives of such parental, and particularly maternal, determinism. Although not yet to the same extremes, the current focus on the first 1,000 days bears unsettling similarities to past narratives. Parents' individual influence over a vulnerable foetus and infant is overemphasized, while the role of societal factors and government responsibility is marginalized. Inadequately supported and poorly contextualized statements for caregivers are found in well‐intended policy and educational materials. A dominant belief is that many of society's most pressing problems can be addressed cheaply and effectively at the individual level. However, such interventions have been disappointingly ineffective. A systemic approach offers a more powerful alternative. Strong evidence underscores the importance of the first 1,000 days, and rather than to use it to reinforce individual blame, it should inform collective action. Knowledge on the importance of the first 1,000 days should drive policies that reduce social inequalities, strengthen parental support structures and create the societal conditions for *all* children to thrive. The first 1,000 days should not be viewed through a deterministic lens. Human development is complex and adaptable, and policies must acknowledge and foster this resilience.

## What should be done to support parents?

We can change the narrative on the importance of the first 1,000 days at different levels: (a) *Research* should continue to explore the interplay between biological, psychological and societal factors in early development. Studies should incorporate a systems lens and include the role of structural societal conditions, such as parental leave policies and access to healthcare, rather than overemphasizing individual parental choices. (b) *Governments* should prioritize investments in a broad range of supportive policies, including paid parental leave, accessible child care, comprehensive prenatal and postnatal support systems, affordable housing and poverty alleviation, ensuring that all families can provide a nurturing environment. (c) *Society at large can* support through community‐based initiatives that reduce isolation, offer practical assistance and acknowledge the diversity of parenting experiences. Public messaging should empower parents rather than burden them with unrealistic expectations.

By fulfilling their obligations under the Convention on the Rights of the Child, governments can reinforce every child's fundamental right to a healthy, secure start in life. By recognizing that parents and children are part of a larger ecosystem, we pave the way for a future where the well‐being of children is a shared priority across all sectors of society. If science, policy and society work together, parenting will no longer be seen as an isolated individual responsibility but will be recognized for what it truly is: a shared societal effort.

## Ethical information

Ethics approval is not applicable.

## Data Availability

Availability is not applicable.
